# Four Major Channels Detected in the Cytochrome P450 3A4: A Step toward Understanding Its Multispecificity

**DOI:** 10.3390/ijms20040987

**Published:** 2019-02-25

**Authors:** Lydia Benkaidali, François André, Gautier Moroy, Bahoueddine Tangour, François Maurel, Michel Petitjean

**Affiliations:** 1 Laboratoire de Biochimie Théorique, CNRS UPR 9080, Institut de Biologie Physico-Chimique, 75005 Paris, France; lydia.benkaidali@gmail.com; 2 CEA/I2BC, CNRS UMR 9198, Université Paris Saclay, 91190 Gif-sur-Yvette, France; francois.andre@i2bc.paris-saclay.fr; 3 MTi, INSERM UMR-S 973, Université Paris Diderot, 75013 Paris, France; gautier.moroy@univ-paris-diderot.fr; 4 Unité de Recherche de Modélisation en Sciences Fondamentales et Didactique, BP244, Université de Tunis El Manar, 2092 Tunis, Tunisie; bahoueddine.tangour@ipeiem.utm.tn; 5 ITODYS, CNRS UMR 7086, Université Paris Diderot, 75013 Paris, France; francois.maurel@univ-paris-diderot.fr; 6 E-pôle de Génoinformatique, CNRS UMR 7592, Institut Jacques Monod, 75013 Paris, France

**Keywords:** cytochromes P450, CYP3A4, active site access channels, cavities boundaries, minimal cost paths

## Abstract

We computed the network of channels of the 3A4 isoform of the cytochrome P450 (CYP) on the basis of 16 crystal structures extracted from the Protein Data Bank (PDB). The calculations were performed with version 2 of the CCCPP software that we developed for this research project. We identified the minimal cost paths (MCPs) output by CCCPP as probable ways to access to the buried active site. The algorithm of calculation of the MCPs is presented in this paper, with its original method of visualization of the channels. We found that these MCPs constitute four major channels in CYP3A4. Among the many channels proposed by Cojocaru et al. in 2007, we found that only four of them open in 3A4. We provide a refined description of these channels together with associated quantitative data.

## 1. Introduction

The cytochromes P450 (CYP) constitute the largest superfamily of hemoproteins, which have been studied since the late 1940s (see [1,2] for an historical survey). With the emergence of genomic data and the quickly growing number of P450 sequences, the superfamily has been phylogenetically classified in families, subfamilies and individuals, respectively, denoted by the first identification number, the letter following it and the second identification number (e.g., in human 1A2, 3A4, etc.). More than 18,000 P450s sequences in all living kingdoms were recognized in 2013 [3], but, since then, the number of known sequences keeps growing and can be estimated to be higher than 300,000 taking into account the current plant genomic projects [4]. CYPs are found in many bacteria, plants and animals. It is estimated that, in human or mammal metabolism, 75% of drug transformation reactions involve catalysis by P450s [5,6]. The secondary and tertiary structures of the CYPs have largely been conserved throughout evolution [7]. The crystal structures of a large number of CYPs, in both the free and the substrate-bound forms, have been solved. The core is highly conserved within the structural fold, and the heminic active site of the CYP is buried inside the enzyme [7].

The human genome encodes 57 CYP isoforms, that play a major role in the biotransformation of drugs, pesticides or many other chemicals, and in the metabolism of endogenous compounds such as steroids and vitamins [8,9]. The detoxification reaction mediated by these CYPs can yield reactive intermediates which can damage DNA, as well as lipids and proteins [10], while the alteration of their activity often leads to serious diseases [11]. Tables of substrates of human isoforms are available [6,12,13,14]. Despite several attempts to predict substrates of CYPs [15,16,17,18,19], no clear prediction rule is known. It is known that lipophilicity can play a crucial role [20] and a summary of substrates/selectivity rules is proposed [21]. However, there is an urgent need to improve the accuracy, interpretability and confidence of the computation models used in drug discovery process (see [19] and references therein).

In this paper, we consider the human isoform 3A4. It lies in the human liver and is estimated to contribute to the phase I metabolism of roughly half of the drugs on the market [22,23]. The other isoforms accounting for more than 90% of the oxidation of drugs are 1A2, 2A6, 2C9, and 2D6 [24]. Although most of the CYPs have a binding allosteric site for their substrates which reversibly accommodates one molecule of substrate at a time, 3A4 isoform can accommodate more than one molecule in its binding site at the same time [25]. Recent advances about CYP3A4 show limited information on the pathways to the heminic site [26,27].

P450s catalyze an oxidation where the substrate binds in the active site on the distal side of the heme. Although the oxidation step has been investigated for a long [28,29,30,31], the ingress and egress of the compounds to and from the active site remain unclear. Structural flexibility is essential to allow chemical compounds to get in and out the active site, and it was shown that it correlates with substrate preferences for several CYPs, including for 3A4 [32].

Few biophysical and biochemical approaches have been proposed by wet biology teams to experimentally address the role of ligand access channels, as reviewed in [33], but never for CYP3A4. To our knowledge, only one article presents clear-cut results suggesting that a ligand diffuses through a given channel and not another one [34]. The authors mutated selected residues in one of the channels (double mutant Y309C/S360C) to introduce cross-linking by disulfide bond that resulted in one channel closure. They could then measure the kinetics of metabolism of two different substrates (benzphetamine and 7-EFC, i.e., 7-ethoxy-trifluoro-coumarine), and show that the double mutant exhibited unchanged activity for benzphetamine (98% of wild type activity), while it dropped to 19% compared to wild-type activity for 7-EFC, indicating that the two substrates do not cross the same channel to access the active site. This experiment necessitates choosing carefully the two residues to mutate, and obtaining an active form of the recombinant enzyme, which is never obvious.

Molecular dynamics based studies of the channels of several CYPs were performed [32,35,36,37,38,39,40,41,42,43,44,45,46,47,48,49,50,51,52,53,54,55]. Some of them were applied to 3A4 [32,42,43,44,45,48,51,53,55]. However, these studies do not lead to a consensus on the number and type of channels for a given isoform. Due to the prohibitively long time scale required to observe opening or closing of channels, we preferred to use a rapid geometric method to identify through which paths are travelling the compounds. Identifying and characterizing the access channels and their lining amino acids is not a trivial task because the channels can dynamically open/close in response to water or ligand passage and enzyme breathing motions [56].

In this context, we computed cavities and channels with CCCPP, which takes in account both the size and the shape of the ligands through a cylindrical model of the ligands, proved to be more realistic than the spherical model used almost everywhere in the literature [57]. The effect of the ligands conformational flexibility on their shape was taken in account in preliminary studies [58,59]. Then, we found that the 3A4 isoform has three major conformations while only two conformations are considered in the literature [60].

For the present study, we built version 2 of CCCPP to perform a refined analysis of the channels. Unless otherwise stated, further mentions of CCCPP refer to its enhanced version. The secondary structure of the members of the P450 family is described by a nomenclature defined by Poulos et al. [61] (see also Figure 1 in [62] and Figure 2 in [63]), which is widely used in the P450 scientific community and that we use throughout this paper. The major description of the channels [64] is based on a geometric method in terms of the secondary structures at which there is an egress of the computed channels. These channels were computed with the software CAVER [65], and gave rise to a channel nomenclature which is still in use [64]. However, there are dozens of other cavities and channels calculation softwares (see [57] for a review). They give a variety of results due to the diversity of output data structures. Thus, it is rather difficult to compare these results. For example, the CAVER based nomenclature established in [64] from all CYP crystal structures available in the Protein Data Bank (PDB) in March 2006 (total: 143 PDB files, 192 chains, 26 CYPs) indicates 14 channels, while CYP3A4 alone (PDB code 1TQN, 1 chain, included in the 2006 study) gave rise in 2012 to 21 channels with MOLEonline 2.0 [66], one of the successors of CAVER. In fact, only three channels were attributed to 3A4 in the 2006 study. Such discrepancies are not unusual. They appeared also between the P450${}_{cam}$ (CYP101) channels calculated with CAVER and MOLEonline and the ones computed in [67], and the situation remains unclear despite the help of several molecular dynamics simulations [35,37]. The experimentalists are left with dozens of published software packages and they have to face to a huge of potentially contradictory results about the channels they are looking for: Which channels should be retained? Easy and rapid comparisons are needed. Giving the name of the secondary structure at which there is a channel egress does not suffice to describe the channels. For a given CYP chain, most of the channels have common parts. Thus, in our opinion, the network of channels should be described with the help of graph theory tools, in terms of paths along nodes and edges, as done in the present study. To compare these networks for different input CYPs, it is better to give a full description of the channels in terms of protein heavy atoms and residues, not only at the egress locations of the channels, but also all along the channels. These functionalities were unavailable in the original version of CCCPP described in [57]. Thus, no more visual examination of the secondary structures is needed to locate the egress of the channels, as it was needed with CAVER. Moreover, the lists of atoms and residues are returned by CCCPP, plus the data structure defining the boundary of each channel. This latter functionality was also available in the version 1 of CCCPP. Throughout this paper, channels named 1, 2a, 2b, etc., refer to the nomenclature of Cojocaru et al. [64] based on the secondary structures elements at the protein surface where the channels emerge.

## 2. Methods

### 2.1. The Standard Approach: Terminology

The channels in proteins were calculated with the CCCPP software (binaries and documentation available at http://petitjeanmichel.free.fr/itoweb.petitjean.freeware.html). The first part of the method implemented in CCCPP is described in [57]. For clarity, we summarize it as follows. The smallest convex domain enclosing the heavy atoms of the protein is a polyhedron partitioned in non overlapping tetrahedral cells with atoms at their vertices (Delaunay triangulation). Two adjacent cells are separated by a triangle with atoms at its vertices, acting as a door between two tetrahedral rooms, which let or not the ligand pass through to travel from one cell to its neighbor. Having flagged all triangular doors with their status, open or closed, it is easy to exhibit the protein shape and its concavities: the protein shape is modelized by the set of tetrahedral cells interconnected by triangles, which can not be passed by the ligand, although the other cells are part of the concavities. Thus, it can be seen whether or not the ligand is sterically allowed to travel from the exterior of the protein to the location of the active site.

It is emphasized that the concavities (or channels) available to the ligand depend on which ligand is considered, and by no way constitute a universal network of concavities (or channels). That should not be shocking: e.g., the space available in the protein to a small molecule such as water cannot be identical to the space available to a large ligand such as cyclosporin or erythromycin.

We also emphasize that the usual terminology dealing with voids inside proteins does not yet make consensus: channels, concavities, pores, pockets, etc. Here, we call channels the concavities linking the exterior of the protein to its buried active site. In the case of a protein with an active site at its surface, we would say that the concavity is a pocket, while surface concavities without any active site are also often called pockets. A concavity throughout the protein and linking its exterior at two places can be called a pore, without reference to any active site. We insist that these intuitive definitions are introduced for clarity but are not intended to be mathematically rigorous.

However, our data structure is rigorously defined and can be handled with graph theory tools. The facial graph was defined as follows: each tetrahedral cell is a node of this graph, and each triangle between two adjacent tetrahedra (i.e., two nodes) is an edge of the graph linking these two nodes if and only if the ligand can pass through this triangle. In general, the facial graph is not connected: it has several components. Any component linking the exterior of the protein to the active site is called a channel. Each ligand has a smallest size (thickness) denoted by CV (critical value) [57]. There is a largest CV for which at least one access channel to the active site exists: it is called the limiting CV, and is denoted CV${}_{lim}$. Above this value, it is declared that the ligand cannot access to the active site due to sterical constraints. The reader is referred to the original paper [57] for advanced technical details.

### 2.2. The Improved Approach: Minimal Cost Paths

The new part of CCCPP that we developed in the framework of the present study is presented below. The full CCCPP software is publicly available on a repository located at http://petitjeanmichel.free.fr/itoweb.petitjean.freeware.html.

It appeared that the channels of the CYPs have large parts at the protein surface and that the main channel to the active site is a funnel which permits several potential pathways for the ligand. To find preferential trajectories for the ligand, we defined a minimal cost path, denoted MCP, as follows. To each edge of the facial graph is associated the cost CV/CV${}_{max}$, where CV is the critical value of the current ligand, and CV${}_{max}$ is the maximal critical value which would allow a hypothetical ligand to pass through the triangle associated to this edge. This cost is in the interval (0,1). The smaller is the cost, easier is the passage. In the facial graph defined in Section 2.1 we can seek for the MCP among all possible paths linking the exterior of the protein to the active site. This is performed with the algorithm of Dijkstra [68]. To detect further potential pathways of interest, all edges of the current MCP are removed, then Dijkstra’s algorithm is applied again, and so on until no new MCP can be found.

Each MCP is an ordered sequence of triangles, but it is also an ordered sequence of tetrahedra. Discarding if it is a channel or a MCP inside a channel, a set of tetrahedra has a volume, which is the sum of the volumes of the tetrahedra. It also has a boundary, which is the set of the triangular faces through which the ligand cannot pass. Thus, it has a surface, which is the sum of the surfaces of these latter triangles. The MCPs are clusterized. Each cluster defines a trajectory: it has surrounding atoms, residues and secondary structures [60]. Here, these trajectories correspond to channels, in the sense of [64].

### 2.3. The Two Modes of Visualization of the Channels and Pathways

**Figure 1:**
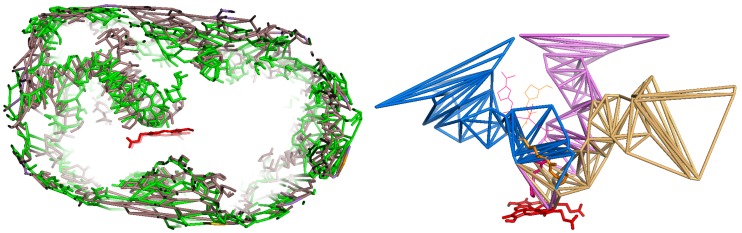
The two basic modes of visualization of CCCPP (images from [69]). The target atom is the iron of the heme group (in red). (**Left**) Superposition of the networks of channels of two complexes of CYP3A4, PDB codes 1TQN and 2V0M, respectively, in green and in brown, computed at CV${}_{lim}$ 6 Å and 7 Å. The edges are those of the facial graph of the pockets and channels: They show the location of the voids in the CYP (it is why most of them lie at the surface of the CYP). (**Right**) The channels 2a (in brown), 2f (in purple) and S (in blue) computed by CCCPP in the complex 4K9U of CYP3A4, a,t respectively, CV = 5.75 Å, 6.25 Å and 6.75 Å. The edges are those of the tetrahedra:They show the boundaries of the channels (they are inside the CYP).

These two modes of visualization are exemplified in Figure 1.

The first mode of visualization of the channels relies on the facial graph of the channels, or parts of this facial graph. It is done by generating a molecular file such that each tetrahedron is a virtual atom located at the barycenter of its four surrounding protein atoms, and the edge connecting two tetrahedra is a bond between their two respective associated virtual atoms.

The second mode of visualization applies mainly to pathways in channels. MCPs can be visualized by generating a molecular file containing the edges of the tetrahedral cells as bonds linking protein atoms. It is pointed out that these bonds originate from the triangulation of the protein, and as such in general they are not chemical bonds between protein atoms: this is just a functionality of CCCPP.

All figures displayed in this paper were generated with the help of PyMOL${}^{TM}$ (The PyMOL Molecular Graphics System, Version 1.2r3pre, Schrödinger, LLC, https://pymol.org/). Some of these figures are based on a mix of the two modes of visualization with appropriate clipping planes, sometimes together with the heme and the ligand.

## 3. Results and Discussion

### 3.1. The Main Channel

**Figure 2:**
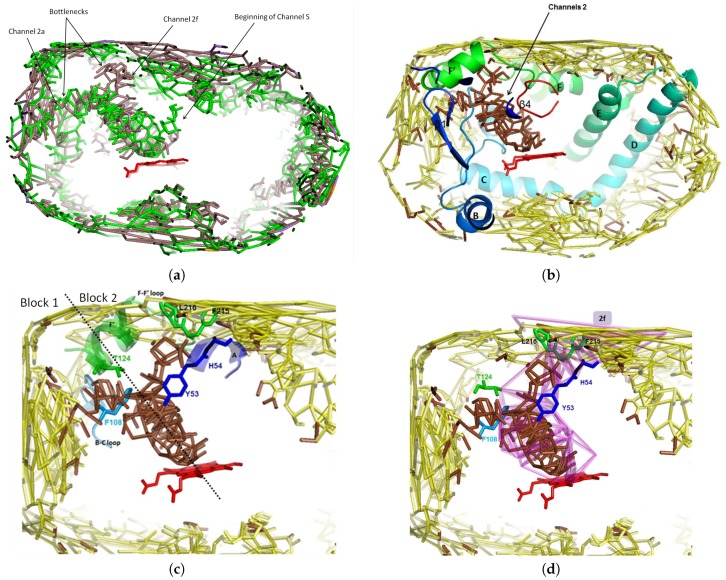
Four representations of the main channel through its facial graph. The target atom is the iron of the heme group (in red). (**a**) Facial graph of the main channel to the active site of CYP3A4 computed from 2V0M (in brown, for CV${}_{lim}$ = 7 Å), superposed to the facial graph of this channel computed from 1TQN (in green, for CV${}_{lim}$ = 6 Å). The two facial graphs show the way in channel 2a: the one of 2V0M is the beginning of channel 2f with a bottleneck at its entry (not connected to the exterior); the two facial graphs show also the beginning of channels S. (**b**) The funnel shaped channel leading to the active site, computed from 2V0M, appearing at CV${}_{lim}$ = 7 Å (in brown) superposed on the one computed at CV = 7.25 Å (in yellow); the part of the facial graph of channels 2 lies within $\beta_{1}$ sheet and C-terminal loop and B-C and F-G loops; only surface pockets were visible for CV > CV${}_{lim}$ (in yellow), thus the evidence of a bottleneck at the entry of the two pathways. (**c**) Zoom of (**b**); bottleneck toward 2a: Phe108 (in B-C loop) and Thr224 (in helix F’); bottleneck toward 2f: Tyr53 and His54 (both in helix A), Phe215 and Leu216 (both in F-F’ loop); the motions of these residues induce the opening/closing of the bottlenecks; the residues constituting the bottleneck are in green and blue sticks; the dashed line separates blocks 1 and 2. (**d**) Zoom of (**b**), superposed on channel 2f computed at CV = 5.75 Å; six residues border the bottlenecks of channel 2f: four are located at the entry (in helix A and FF’ loop), Phe108 in the common part of 2a and 2f, and Thr224 where 2a and 2f separate.

A funnel shaped channel appears in the apo form 1TQN of CYP3A4 (Figure 2a). It is located in the deformable area of channel 2 (as named by Cojocaru et al. [64]) and lets a wide opening at the neighbor of the active site. It lies between the following secondary structures: $\beta_{1}$ sheet, A-anchor, B-C block and $\beta_{4}$ sheet (in 3A4, it is the C-terminal loop, as denoted further in the text; size slightly depending on the conformations of a given isoform), and F-G block (helix-loop-helix), very flexible due to channel opening (Figure 2b). The B-C block is defined to extend from the beginning of the B helix to the end of the C helix, and the F-G block to extend from the beginning of the F helix to the end of the G helix. The F-G block region offers highly variable amino acids sequences and structures for different CYPs, and thus can be important for substrate specificity through making contacts with the substrate. These regions are bordered by putative SRSs (Substrate Recognition Site: see [70]). The flexibility of the F-F’ loop let us define three conformations of CYP3A4 [60]: the closed one, labeled C, and the opened conformations, labeled O1 or O2 depending, respectively, if block 1 or block 2 opens. Molecular dynamics simulations of cytochrome P450${}_{cam}$ showed that substrates and products could egress from the active site via pathways in the vicinity of the three routes identified by TMP (thermal motion pathway) analysis, but with pathway 2 being energetically favored over pathways 1 and 3 [36,37]. Analysis of the simulations of cytochromes P450${}_{cam}$, P450-BM3 and P450${}_{eryF}$, showed that egress trajectories in the region of channel 2 could be clustered into subclasses, named 2a, 2b, etc., according to the secondary structure elements lining the ligand pathway as it emerges from the protein surface. Although the overall fold of the CYPs is well conserved, the length and secondary structure of the B-C and F-G blocks vary considerably inside the P450s family. The flexibility of these 2 blocks is the main source of conformational change for a given isoform [32].

The facial graph (see Section 2.1) of this main channel is connected, i.e., there is only one channel there: see Figure 2a,b. Knowing the volumes of the tetrahedra issued from the triangulation, we computed that about 88% of the void appears to be surface pockets and only 12% of the void is located at the distal face of the heme (Figure 2a,b). Some ligands are in surface binding pockets, such as the progesterone [71] (PDB code 1W0F; see also Figure 4 in [57]). It could be considered that surface pockets are concavities which are not part of the protein domain, but there is no consensus in the literature about the definition of the protein domain and of the pockets. The binding cavity has a large volume, reported to range from 1173 to 1332 Å${}^{3}$, increasing up to 2000 Å${}^{3}$ when binding large substrates such as ketoconazole and erythromycin [72]. The whole channel 2 computed by CCCPP for CV = 6 Å (see the definition of CV in Section 2.1), which includes the binding cavity, the path accessing to it and the mouth of the channel, has a total volume of 42,400 Å${}^{3}$ and a bounding surface of 41,800 Å${}^{2}$. These values are large because they include the contribution of the mouth of the channel, which is a large cavity lying at the protein surface. It is pointed out that the status of such a surface cavity is unclear because, while it is inside the convex hull of the protein, it is difficult to decide if it is indeed a part of the protein domain or if it is a void region exterior to the non convex shaped protein.

The holo form 2V0M of CYP3A4 contains two ketoconazoles molecules. This antifungal molecule is bulky (van der Waals volume: 450 Å${}^{3}$). The funnel appears for a maximal ligand size CV${}_{lim}$ = 7 Å (see the definition of CV${}_{lim}$ in Section 2.1), and leads to observe two pathways, each corresponding to a subchannel and containing one ketoconazole. These two subchannels are in block 1 and block 2 [60]. In the apo form, only block 1 appears to let pass the first ketoconazole (Figure 2a). In the holo form, block 2 is opened at CV = 5.75 Å because its access is obstructed by a bottleneck of CV < 6 Å (see Figure 2c). At this CV, block 2 appears (Figure 2a).

**Figure 3:**
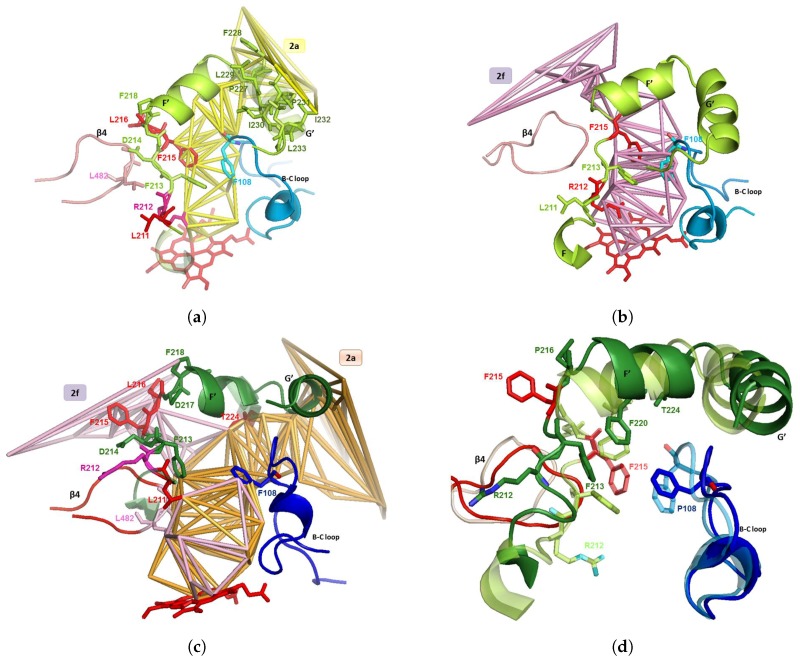
Apo forms are in light colors, holo forms are in dark colors. Each holo form refers to 2V0M, with two ketoconazoles (see Figure 5). (**a**) Channel 2a in 1TQN with some of its bounding residues in the F-F’ and B-C loops; the mouth of the channel is lined by G’ helix hydrophobic residues; the gating residues Phe213 and Phe215 of the F-F’ loop and Phe108 of the B-C loop are bounded by channel 2a in closed form. (**b**) Channel 2f of 2V0M, superposed on 1TQN; shows the steric obstruction of lining residues of F-F’ and B-C loops (Arg212 and gating residues Phe213, Phe215 and Phe108) and of helix F’ to open channel 2f. (**c**) Channels 2a and 2f in 2V0M with some bounding residues; the common part of channels 2a and 2f is lined by F-F’ and B-C loops and by C-terminal loop; the mouth of 2a is lined by helix G’ helix and by the mouth of 2f by F-F’ loop; 2a and 2f are separated at exit by hydroxy-Thr224 in helix F’ at the hydroxy group; the common part is in orange and the separated parts are in light rose; the gating residues Phe 213 and Phe215 in loop F-F’ and Phe108 in B-C loop borders 2a and 2f in the open form of CYP3A4. (**d**) 1TQN superposed on 2V0M; the secondary structures are in cartoon and the residues are in stick (gating ones: Leu216, Arg212); 1TQN is transparent, showing 3A4 conformation before entrance of the two ketoconazoles; indicate what could be the moves of B-C and F-G blocks and of C-terminal loop, involved in the opening of access channels 2a and 2f.

Several crystallographic structures of CYP3A4 exist, in several conformations corresponding to different states: interaction with one or two ligands, or none. These differences are related to conformational changes, which can be correlated to the differences between the channels, in function of the ligand. To accommodate two molecules or a large molecule, the protein undergoes a significant conformational change, especially in the F-G region and around the Phenyle cluster, which contains eight phenylalanines residues (57, 108, 213, 215, 219, 220, 241 and 304). Positioning of the I-helix and the C-terminal loop are also altered. The apo form 1TQN is flagged as closed subtype 1 [64,73]. It is such that the F-F’ loop binds the ligand in 2V0M (Figure 3c), and is flagged as open subtype 2 [60,64,73]. It is bound to two ketoconazoles, a known inhibitor of CYP3A4. Ketoconazole has an imidazole group (highly polar) and it has nine rotatable bonds and thus it is highly flexible [59]. In 2V0M there are two co-crystallized ketoconazoles (respective thicknesses of 5.19 Å and 6.52 Å, measured as indicated in [57]).

**Figure 4:**
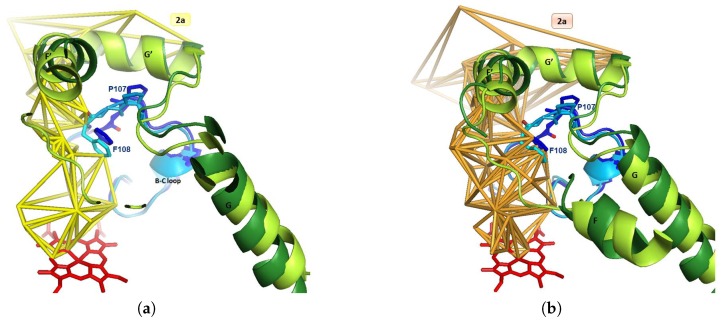
Superposition of CYP3A4 1TQN and 1V0M at blocks F-G and B-C, which bound channel 2a. For clarity, a piece of F-F’ loop was removed. Apo forms are in light colors, holo forms are in dark colors. Each holo form refers to 2V0M, with two ketoconazoles (see Figure 5). (**a**) Channel 2a of 1TQN (CV = 6 Å) superposed on 2V0M. (**b**) Channel 2a of 2V0M (CV = 7 Å) superposed on 1TQN.

The first one has its imidazole group near the heme: the N3 atom of the imidazole ring binds the iron atom of the heme [74]. The dichlorinated aromatic ring is in the active site and the main skeleton is in the part of channel 2a common with channel 2f (channels 2a and 2f separate at Thr224). The bottleneck in channel 2a is at Phe108 and Thr224. Compared to the apo structure, an enlargement of channel 2a of 1 Å is needed to accept a large ligand (Figure 3c and Figure 4a,b).

**Figure 5:**
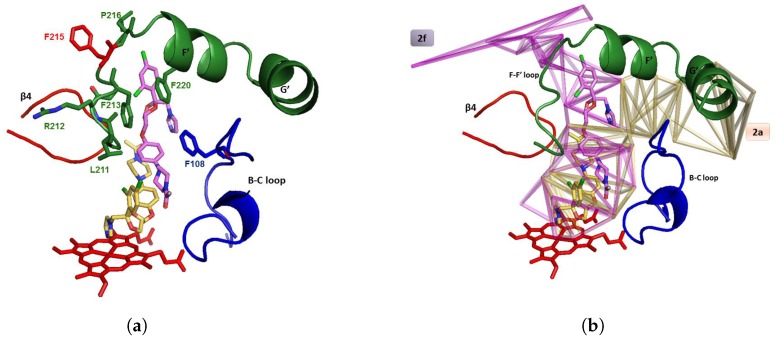
(**a**) The two ketoconazoles of 2V0M inside channels 2a and 2f, within the secondary structures bounding these channels (these latter are not delineated). The first ketoconazole, in yellow, enters through channel 2a. The second ketoconazole, in purple, enters through channel 2f. (**b**) Idem, with delineation of channels 2a and 2f by the boundaries of the trajectories (residues are not displayed).

The second ketoconazole is inside channel 2f with an orientation opposite to the one of the first ketoconazole, and with the acetyl group near the active site and the dichlorinated aromatic ring (more hyrophobic) near the entry of the channel (Figure 5a,b). The second ketoconazole is inside channel 2f but it has the opposite orientation, with the acetyl group near the active site and the dichlorinated aromatic ring (more hyrophobic) near the entry of the channel (Figure 5a,b). There is a bottleneck in channel 2f, surrounded by four residues (Tyr53, His54, Phe215, and Leu216), three of them being aromatic and acting as gating residues: see Figure 3b. Phe215 and Leu216 are in F-F’ loop after conformational change of the apo structure where Phe215 obstructed the opening of channel 2f. Tyr53 and His54 are on anchored helix A in the membrane without significant change from the apo structure, and are thus assumed to have no role in building the bottleneck. Thus, this latter would be due only to a move of the F-F’ loop. Phe215 moves in the holo structure toward the mouth of channel 2f and catches the hydrophobic ligand at the membrane/water interface: this is a difference with the first molecule, which enters in channel 2a through the membrane, thus explaining the opposite orientations of the two molecules. The movement of F-F’ loop let Arg212 go to the interior of the protein at a location at the opposite of the active site: Arg212 is no longer able to interact with the ligand.

### 3.2. Access Channels and Narrow Channels

#### 3.2.1. Channels 2a and 2f

We know that the access to 3A4 active site is through channel 2a, computed from the apo structure with CV = 6 Å. This channel is opened in the apo form, and it enlarges to accept a first ketoconazole ligand at CV = 7 Å between the F-G and the B-C loops and the $\beta_{1}$ sheet. Channel 2a is the first found by CCCPP, and it is the biggest one for this conformation of CYP3A4. It contains the first ketoconazole, bound to the heme. This channel is assumed to be the first pathway found by the ketoconazole. Pathway 2a, in which the ligand passes between F-G loop, B-C loop and the $\beta_{1}$ sheet, appeared to be the most likely route for substrate access and product egress in previous study on bacterial proteins [75]. In the bacterial P450 structures, 2a is opened in most of the structures that have at least one channel opened. Simulations of product egress from CYP101 indicate that this route would be used by the product as well as the substrate [36]. The MCP (see Section 2.2) corresponding to channel 2a has a volume of 613 Å${}^{3}$ and a bounding surface of 273 Å${}^{2}$ in the apo form, and it has a volume of 774 Å${}^{3}$ and a bounding surface of 896 Å${}^{2}$ in the holo form.This volume is small because it corresponds to the path accessing to the binding site rather than to the full binding cavity. It should be compared to the volume of convex hull of the ketoconazole, which is estimated to be in the interval 300–340 Å${}^{3}$, depending on the conformation. The surface boundary is small because the path is not a closed one: it is bounded only at some places while it opens on the rest of the channel at other places. Such quantitative results got by CCCPP cannot be obtained by other channel calculation programs.

Then, other pathways are needed to accept more ligands. In the holo form, a second channel is found: it is channel 2f, in block 2, located between F-G block and C-terminal loop, and containing the second ketoconazole. It is identified as distinct channel and it lies between channel 2a and the solvent channel S. In our study of the 16 crystallographic structures, we observe that the channel 2f opens for CV in the range 5.75–8.75 Å, depending on the ligand size. In 2V0M, channels 2a and 2f have a common part and separate at exit in helix F’ at hydroxy group of Thr224. We found that the second ketoconazole of structure 2V0M is oriented from the heme in the direction of channel 2f. At CV${}_{lim}$ = 7 Å, channel 2f appears as a dead end, not connected to the exterior (Figure 2a). It has a total volume of 49,200 Å${}^{3}$ and a bounding surface of 48,100 Å${}^{2}$. The MCP in channel 2f has a volume of 851 Å${}^{3}$ and a bounding surface of 360 Å${}^{2}$ in the holo form. A bottleneck at CV < 6 Å obstructs the connection to the exterior of the protein. An opening at CV = 5.75 Å and a slight channel flexibility should permit the second ligand to enter (CV = 6.52 Å). Channels 2a and 2f are bordered by SRS-2, lying above the active site cavity.

**Figure 6:**
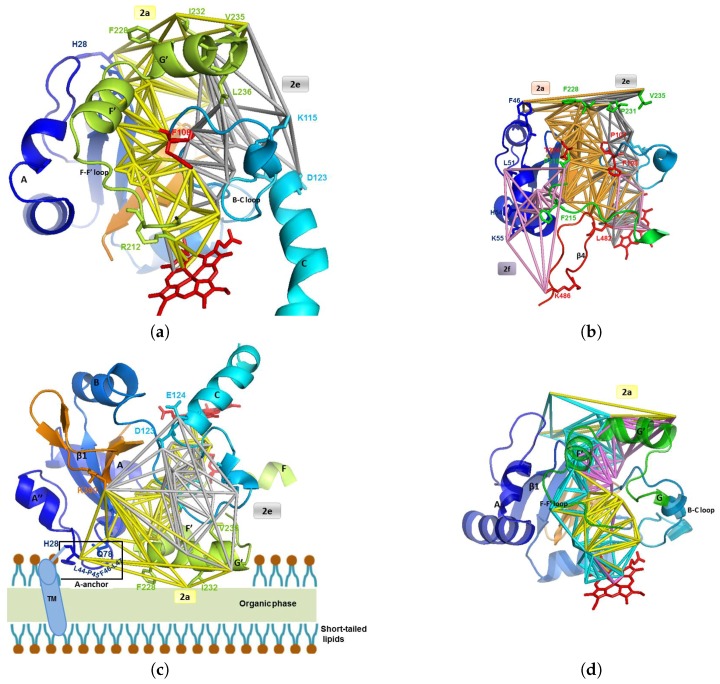
(**a**) Channels 2a and 2e within lining secondary structures of 1TQN ($\beta_{1}$ sheet, F-G and B-C blocks): the two mouths are neighboring, then the channels separate at Pro107 and Phe108 in B-C loop. (**b**) Channels 2a, 2f and 2e of 2V0M enclosed by $\beta_{1}$ sheet, F-G and B-C blocks, and C-terminal loop; channels 2a and 2e are separated at Pro107 and Phe108 in B-C loop. Channels 2a and 2f are separated by Thr224 in helix F’. (**c**) Same as (**a**), rotated: the bottom part of this figure is a part of Figure 1 in [27] (©American Chemical Society, https://pubs.acs.org/doi/abs/10.1021%2Fja4003525). Membrane-bound form of CYP3A4 shows the location of the mouths of 2a and 2e in 1TQN and highlights residues inserting into the membrane and interacting with it via the hydrophobic side chains of helices F’ and G’ and of A-anchor. A-anchor has a fixed position on A’A" loop, and is formed by residues His28, Leu44, Pro45, Phe46, Leu47 and Gln78: it is labeled in dark blue and boxed. Channel 2a leads to the membrane; channel 2e leads to cytosol; the membrane is viewed on the side of the globular domain; the transmembrane helix is visible; and the location of the organic phase and the short-tailed lipids are represented. (**d**) Channel 2a represented by three MCPs, respectively, in purple, yellow and cyan, surrounded by secondary structures. The three MCPs have a common part in channel 2a.

Channels 2a and 2f are hydrophobic. They have a common part then separate near the hydroxy group of Thr224, on helix F’ (see Figure 2c). Channel 2a is bordered by SRS-1, SRS-2 and SRS-3, and channel 2f is bordered by SRS-2 and SRS-6. Both channels 2a and 2f open at the C-terminal region of the helix F and in the F-F’ loop, forming SRS-2 (near the active site cavity). The other part of the entry of channel 2a lies at the N-terminal region of the helix G and in the G-G’ loop, forming SRS-3 (see Figure 5a). The B-C loop/B’ helix (bordering channel 2a) was identified as a putative SRS-1 [70], and involved in substrate binding. We found with CCCPP that the opening of channel 2a is apolar for 1TQN, located at His28, Phe228, Ile232 and Val235. It is more polar for 2V0M, located at Lys35, Phe46, Phe228, Val235, Asp380, and Lys390. For both 1TQN and 2V0M, the entry of 2a is bordered on one of its sides by the hydrophobic helix G’ (residues 230–237; see Figure 6a,b), which is anchored in the membrane (see Figure 3 and Figure 6a,c). The following residues of channel 2a have an orientation common to the apo form 1TQN and to the holo form 2V0M: Pro227, Phe228, Leu229, Ile230, Pro231, Ile232, Leu233, Glu234, Val235, and Leu236. Channel 2a enlarges of ΔCV~1 Å and channel 2f opens to accept two ketoconazoles in 2V0M. The superposition of 1TQN and 2V0M shows that the two B-C loops are superposed due to a move of B-C in 2V0M at Phe108, Gly109, and Pro110, at the exterior of the funnel area, so that it permits the entrance of the ligand in 2a and 2f. These two channels are bordered by par the B-C loop (see Figure 3a,b). In the apo form, Phe108 obstructs the channel. Its move enlarges the common space admitting the two ketoconazoles. In 2V0M, R106 (in B-C loop) and E374 are lining channel 2a. Tyr53 and His54 are on the anchored helix A, without change between the apo form 1TQN and the holo form 2V0M. These few flexible two residues are not involved in the move of the bottleneck of channel 2f. This move is thus due to the move of F-F’ loop. The two bottleneck residues are at the mouth of 2f: Phe215 and Leu216, after the conformational change of F-F’ where Phe215 obstructed the opening of 2f in the apo form 1TQN (Figure 2b).

F-F’ loop is hydrophobic and has three charged residues: Arg212, Asp214 and Asp217. Its move may act as a closing/opening valve, at Leu211, Arg212, Phe213, and Phe215 (Figure 2a,c). F-F’ loop in a closed conformation is exposed to block 1. Its residues Arg212, Phe213 and Phe215 border the body of the hydrophobic channel 2a in the apo form 1TQN. In this latter, only one polar residue, Arg212 (oriented toward the active site: Figure 2a) borders channel 2a. Arg212 was suggested to regulate solvation of the active site [61], and was reported to assist the binding of ligands to the active site [76,77]. Phe215 is in F-F’ loop, bordering channel 2a in conformation C (Figure 2a). Phe215 is involved in the substrate orientation within CYPs active sites and in catalytic mechanisms of these substrates [78]. In the 2V0M, F-F’ loop is distorted to open channel 2f. The orientation of the F-F’ loop residues follow either the same direction (K208, K209, L210, snf D214), the opposite direction (Leu211, Arg212, Phe215, and Leu216), or are oriented in the same sense but shifted (valves: Phe213, Asp217, and Pro218). The opening of channel 2f is at Leu51, His54, Lys55, Phe215, Pro218 and Lys486. Helix F’ borders channel 2f with apolar residues Leu221, Phe220 and Thr224. In the holo form, an opening occcurs via the move of the F-F’ loop such that Phe215 (bordering 2a in the apo form), is pushed toward block 2. This push of Phe215 toward the surface is useful: (i) to suppress the sterical hindrance to free space to open channel 2f and enlarge the cavity near the part common to 2a and 2f; and (ii) to border the entry of channel 2f, as Leu216 goes to the mouth of 2f at the water interface of the membrane/cytosol to catch the second hydrophobic molecule (see Figure 2c). The same phenomenon is observed in the structure 4K9U, which welcomes two molecules of GS5 (a ritonavir analog [79]), with Log(P) = 3.06. This phenomenon is observed neither for 4K9T, which welcomes one GS4 molecule (another ritonavir analog [79]), nor for 2J0D, which welcomes the bulky erythromycin molecule. For these two more hydrophilic molecules (Log(P) = 2.22 and 2.60, respectively), Phe215 is deformed without being located on the mouth of the computed channel 2f. Pro218 remains at the surface and borders the mouth of channel 2f: that lets the mouth of channel 2f more hydrophobic, thus it helps to catch the hydrophobic ligand. Asp217 is at the entry of block 2 but is not exposed at the surface, i.e., it does not border the mouth of 2f, at the opposite of Pro218, which is hydrophobic and borders the mouth of 2f. The residues rearrangements occur with the move of Leu211 (inside the protein in the apo form), to become (in 3A4-ketoconazole complex) oriented in the active site, bordering the common part of channels 2a and 2f, near the active site, at the opposite of Arg212 which is pushed on the other side of channels 2a and 2f (Figure 2c and Figure 3a). This orientation can be correlated with the fact that substituting Leu211 by a phenylalanine residue affects the homotropic cooperativity in the binding with testosterone [80]. The presence of Leu211 compensates the hydrophobicity of channels 2a and 2f after departure of Phe215. Other significant changes are a shift of 4 Å of the C-terminal loop and a deformation of the of the crevice of helix I around Tyr307 (the C${}_{\alpha }$ is shifted at more than 2 Å) [81]. C-terminal loop becomes closer to the common body of the two channels 2a and 2f delineated by Gly481 and Leu482 near the active site (Figure 2a,c).

#### 3.2.2. Detection of Narrow Channels 2e and S

**Figure 7:**
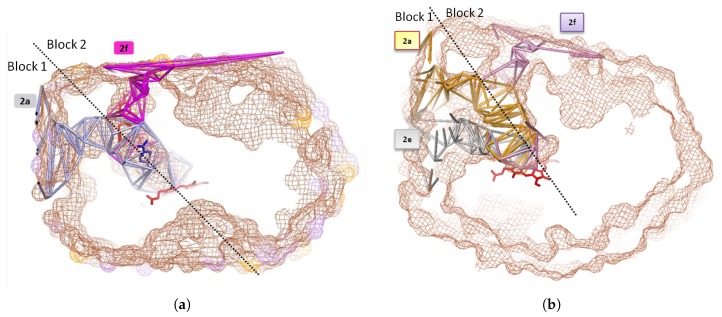
Superposition of the channels of 2V0M. The dashed lines separate blocks 1 and 2. (**a**) Channels 2a and 2f computed at CV = 5.75 Å. (**b**) Channels 2a, 2f and 2e, computed at CV = 5.50 Å.

The narrow channel 2e appears at CV = 5.75 Å in 1TQN and at 5.5 Å in 2V0M. It appears with a total volume in the apo form of 44,500 Å${}^{3}$ (bounding surface: 44,100 Å${}^{2}$) and a volume in the holo form of 51,000 Å${}^{3}$ (bounding surface: 48,200 Å${}^{2}$). It is a rather common channel observed in twelve different crystallized P450 isoforms [64]. This channel 2e was observed in the 16 crystallized 3A4 structures (available in the pdb data bank) considered this study (see Table 1 in Section 3.3). It has been observed that the channel 2e opens for CV in the range of CV between 5.5 Å and 6 Å, without any relationship with the fact that the protein is bound or not. Channel 2e appears for all conformations of CYP3A4 (C, O1, O2). It threads through the B-C loop (Figure 6), and its opening could depend on the length and of the flexibility of this loop. In the stage of the reaction where these structures of CYP3A4 were crystallized, channel 2e is opened in this range of ΔCV = 0.5 Å, not containing ligand. Channel 2e is detected as a secondary egress route for substrates or products in molecular dynamics simulations of P450s [64]. It is also observed as a secondary exit pathway in simulations of P450eryF [75] and CYP2C5 [40]. It is difficult to conclude about the role of channel 2e as an egress route as long as there is no known structure of complexes with oxidation products. MD (Molecular Dynamics) studies give information on the dynamics of ligand tunnels (opening/closure), but do not involve simulation of the process of ligand egress. MD and SMD (Steering Molecular Dynamics) studies mainly focused on ligand preferred exit tunnel. It was found that channel 2e is an exit one for the hydroxylated product of diazepam in 1TQN, and that channel S is an exit one for 6-hydroxytestosterone [44,45]. That suggests the enlargement of the small opened channel 2e for ligand exit. As shown in Section 1, a controlled hydration of the substrate bound P450 active site is extremely important for catalysis. A solvent filled channel from bulk solvent to heme let water circulate as a water pump. It is likely that its function is related to active site hydration, although it may also have a role in proton transfer (furthermore channel 2e opens on the cytosol). It was suggested that a water channel exists between B-C loop and the C-terminal part of helix B [72]. Channel 2e is displayed with channels 2a and 2f in Figure 7.

In the apo form, channel 2e exits at Lys115, Asp123, Glu124, Pro231, Val235 and Lys390, and channel S exits at Phe22, Pro23, Val235, Asp380 and Lys390. Channels 2a and 2e have a common part near the active site, then separate (Figure 6a,b). Channel 2e and S exit in the cytosol. The residues of the mouth of channel 2e are rather polar, while those of 2a and 2f are apolar (Figure 6c). For the 16 complexes considered in this study, the ligands were located in channels 2a, 2f and S, but none was found in channel 2e. It suggests that channel 2e could be an exit channel, either for oxidized products or for water or protons. Channel 2e may also be for dropping water out of access channels to free space for ligands.

In the apo and holo forms, channel 2e is opened simultaneously with channel 2a, 2f and S, suggesting that the access channels may alternatively open by a F-G move away from the B-C loop, without affecting the 2e channel. This is in agreement with a study on the structure of CYP2C5 [82].

A contiguous water channel from the bulk solvent to the active site is possible [83]. Channel S, i.e., the solvent channel, is detected at CV = 5 Å in the apo form 1TQN. Then, it was computed at CV = 5.75 Å (slightly larger), as a host channel for one of the two GS5 molecules (a ritonavir analog) in the 4K9U complex [79]. It is flagged as important in several isoforms in [64,84,85]. It faces the cytosol and contains a charged gating residue which could lead the product out of the CYP3A4 active site [44]. MD simulations of expulsion of temazepam and 6β-hydroxy testosterone out of CYP3A4 were done: channel S has the largest opening for these two products, so that it may be an exit way for the substrate [44]. Channel S was proposed as a route for controlling water access and egress to the active site for water based on its observation in P450-BM3 [84]. It may also be used for substrate egress [84,86]. It was proposed as the main gateway to the active site of the human 2D6 [86].

#### 3.2.3. Channels Opening/Closing and Interactions with the Lipid Bilayer

In a recent paper [87], it is stated that access channels to the buried active site control substrate specificity in CYP1A P450 enzymes. Then, in a recent review [33], it is suggested that the network of channels is involved in the control of the P450 enzymes substrate specificity for all P450 family. The diversity and the deformability of the channels could explain the diversity of its substrates. We emit the assumption that the specificity of the CYPs relies on what happens at the entry of the channels and that, due to sterical constraints, the orientation of the ligand does not change until it reaches the active site. It was suggested that the channels are often gated by aromatic residues all along them [73]. It was also suggested from the analysis of crystal structures that aromatic residues can form a network of gates, which regulates cooperatively the opening and closing of different tunnels [88]. Except Tyr53 for channel 2f, the following gating residues are phenylalanines:

- channel 2a: 57, 108, 213, 215, 228, and 304.
- channel 2f: 46, 57, 108, 213, 215, 220 (on helix F’), 226, and 304.
- channel 2e: 108, 213, 215, 228, and 304.
- channel S: 108, 213, 220, and 304.

Mammalian CYPs are generally membrane-bound proteins [89,90]. The mechanisms of substrate access and product egress from the mammalian membrane bound P450s may differ from those in the soluble bacterial P450s studied before [53]. The microsomal CYPs are anchored in the membrane by an N-terminal transmembrane alpha-helix and there is evidence that their globular domain dips into the membrane [91]. A membrane bound model of human CYP3A4 provides the structure of the protein membrane complexes consistent with most experimental data [51]. Membrane binding of the globular domain in CYP3A4 significantly reshapes the protein at the membrane interface, where most channels open, inducing conformational changes relevant to access tunnels [27].

The CYPs substrates are rather hydrophobic and in the case of membrane bound P450s they are expected to come from the lipid bilayer. As the products of the P450 catalyzed reactions are more hydrophilic, they may be released into the aqueous environment or the polar headgroup region rather than back into the lipid bilayer. The multiplicity of channels suggests possibilities for ligand channelling to and from the P450s sitting in or on the membrane. The P450 protein topology favors channel formation on the distal side of the heme. The proximal side is the likely reductase binding site and corresponds to the smallest channels found by CCCPP.

Helices F’ and G’ do not completely insert into the membrane, with helix G’ establishing a closer contact to the membrane than helix F’. CYP3A4 is anchored into the membrane helix G’, which partitions mainly within the headgroup region [27]. The mouth of channel 2f is bordered by helix F’ at the water/membrane interface. It was assumed from modeling studies that channel 2f opens at arrival of the molecule [92,93]. Channel opening was observed as a consequence of ligand-induced conformational changes [94]. The mouths of channels 2f that we computed have hydrophobic residues in F-F’ loop: Phe213, Phe215, Leu216 and Pro218; thus, the interaction with the channel mouth is facilitated for hydrophobic molecules.

Given the dynamic nature of membrane-anchored CYPs, the precise positions of channels may change dynamically over time [93]. We retained for our calculations the positioning used in [27].

The dynamic motions of the protein can cause the opening of channels not seen in the crystal structures as well as changes in the relative dimensions of the channels [36,40,75]. Even though these motions cannot be seen dynamically in the crystal structures, the location of the channels in these latter, supported by their capacity of ligands, provides a useful basis for exploring ligand access and egress routes, particularly when the snapshots from different crystal structures are considered together.

The mammalian CYPs are characterized by a subdivision of their larger F-G region in F’ et G’ helix [51]. Insertion of F’-G’ helix-loop of CYP3A4 in the membrane moves the β domain towards the heme plane, allowing channel 2a to open, whereas this opening does not occur in soluble bacterial P450s. In these latter, the beta domain plane is farther from the heme plane: the opening occurs between F-G loop and B helix [26], at the level of the opening between F-F’ loop and the C-terminal loop in 3A4, corresponding to the channel 2f. The opening of block 2 (due to the move of F-F’ loop), which characterizes 3A4, offers a more diverse exterior environment for the compound than the prokaryotic CYPs which offers only solvent exterior channel 2a as environment [36,75].

### 3.3. Characteristics of the Four Major Channels

**Figure 8:**
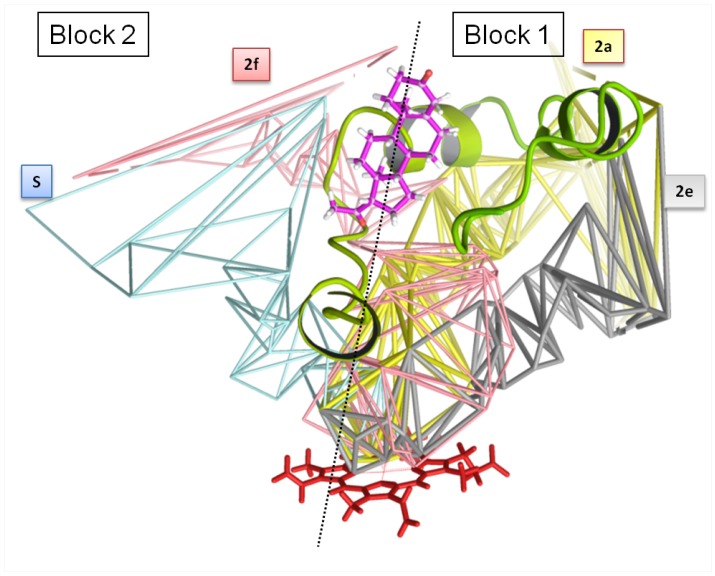
Summary of the four channels of CYP3A4. The heme is shown at the bottom of the channels. The F-F’ loop is in green. The progesterone molecule (in purple) lies in a surface pocket (see also Figure 4 in [57]). The dashed line separates blocks 1 and 2.

For convenience, we summarize these characteristics in Table 2. The four major channels contains substrate recognition sites (SRS). We also summarize in Table 1 the characteristics of the channels computed by CCCPP for the crystallographic structures of CYP3A4 considered in the present study. We recall that CV is the value of the channel bottleneck (see Section 2). When the critical thickness CV of a ligand exceeds CV${}_{lim}$, the bulky part of the ligand is not in the bottleneck: the passage of the ligand requires flexibility. We also recall that the topology of the channels may be constituted by several MCPs having one or several common parts. This was observed for 2J0D and 4K9T, but for clarity in these cases we provide only the CV values of the main paths in Table 1. We show the four channels in Figure 8 and we summarize the location of the four channels as follows: 

In block 1:

- 2a: main channel, apolar mouth (helices F’ and G’), opening in the membrane, hydrophobic ligands.

In block 2:

- 2f: alternate channel, mouth opening in transmembrane region, more hydrophilic ligands.
- S: alternate channel, may be an egress channel, mouth opening on cytosol, polar environment, accepting less hydrophobic molecules.

Neither in block 1 nor in block 2:

- 2e: may be an egress channel, mouth opening on cytosol, apparait at CV${}_{min}$ ~5.5 Å (narrow), exists in all structures in the three conformations, does not contain a molecule.

## 4. Conclusions

Our analysis, carried out with CCCPP program on several crystal structures of CYP3A4, enabled identifying relevant ingress/egress channels, with their lining heavy atoms and residues. Our calculations support the hypothesis of channels 2a and 2f as major channels of substrate/product egress in CYP3A4, plus two secondary ones, 2e and S. We propose potential pathways of the ligands, inside these channels, toward the distal face of the heme together with information on the movements of the residues associated to the opening/closing of the channels.

Our analysis suggests that block 1 anchored in the membrane opens at ligand entrance and channel 2a is the only ingress/egress channel. Then, in the case of one large ligand or two ligands, block 2 opens. Smaller channels 2e and S could be involved in the egress of the metabolites, either by enlarging or by use for circulation of water or dioxygen. We did not consider proximal channels smaller than distal channels.

Channels 2a and 2f are occupied by ligands that are not yet oxidized. Either channel 2a enlarges to accept the ligand, or a new path (channel 2f) opens due to the nature and/or the large size of the ligand [60]. Residues obstructing the channel create a bottleneck. These changes are influenced by the location of the channels with respect to the protein topology and the protein’s overall global motion [37,38]. Channel 2e exits near the cytosol (see Figure 6 in [27]). Channel 2a is opened in the apo structure, although channel 2f is not. At input of the ligand, channel 2a enlarges until reaching a critical value, above which other paths are needed to accept the entrance of more ligands. When the second ligand enters in channel 2f, its orientation is the opposite of the one of the first ligand, thus fitting the weaker hydrophobicity of channel 2f which starts at the cytosol/membrane interface. These movements are located at flexible secondary structures such as B-C and F-F’ loops and C-terminal loop [64]. Molecular dynamics simulations could help to know if channel 2f is closed in the apo form then opens to accept the ligand, or if it is already opened. The F-G block acts as a multi-hinged lid on the distal side of the protein and many channels border it, permitting an opening at the membrane’s interface or on the cytosol.

The major channel lining residues mentioned in our study, which we suggested to have a role in channel opening, were proved to be involved in ligand binding, in the activity or cooperativity of CYP3A4 and to be key residues governing allosteric processes in P450 catalyzed substrate oxidations [92]. Another important structural element is the B-C loop, which borders channels 2a and 2e.

Although molecular dynamics simulations were able to exhibit channels not visible by a rough examination of crystallographic structures [94], it was possible to detect such channels by geometric methods because the ligands were indeed present there. A major use of our results could be to provide pertinent starting points for molecular dynamics computations to observe the opening and closing of the channels.

## Figures and Tables

**Table 1:** The four channels of CYP3A4 and its conformational states (C, O1, O2). The first channel appears at CV${}_{lim}$ and the next channels appear at CV < CV${}_{lim}$. ${}^{a}$ Critical thickness of the ligand in Å computed as in [57]. When there are two ligands, two CV values are reported. ${}^{b}$ Cf: conformation (C, O1, O2), according to the authors of [60,69]. ${}^{c}$ See Section 2. ${}^{d}$ Third channel and eventually fourth channel. For 4K9U, the same CV was observed for the third and the fourth channels. ^e^ Ritonavir analog: see [79]. ${}^{f}$ Other ritonavir analog: see [95]. ${}^{g}$ The topology is constituted by several MCPs having common parts (see Section 2.2).

PDB	Ligand	CV ${}^{a}$	Cf ${}^{b}$	First	CV${}_{\lim}$ ${}^{c}$	Second	CV ${}^{c}$	Next ${}^{d}$	CV ${}^{c,d}$
Code				Channel		Channel		Channels	
1TQN			C	2a	6.00	2e	5.75	S	5.00
1W0E			C	2a	5.75	2e	5.75		
1W0F	Progesterone	4.10	C	2a	6.00	2e	5.75		
1W0G	Metyrapone	4.19	C	2a	6.50	2e	5.50		
3UA1	Bromoergocryptin	6.06	C	2a	6.75	2e	5.50		
4K9V	GS6 ^e^	5.53	C	2a	6.50	2e	6.00		
3NXU	Ritonavir	7.37	O1	2a	6.25	2e	5.75		
4I4G	GS2 ${}^{f}$	6.54	O1	2a	6.00	2e	5.75		
4I4H	GS3 ${}^{f}$	6.72	O1	2a	6.25	2e	6.00		
4K9W	GS7 ^e^	6.47	O1	2a	6.00	2e	5.50		
4K9X	GS8 ^e^	4.77	O1	2a	6.00	2e	5.75		
2J0D	Erythromycine	7.16	O2	2f	8.75	2f ${}^{g}$	6.75	2e	5.50
2V0M	Ketoconazole	5.19, 6.52	O2	2a	7.00	2f	5.75	2e	5.50
4K9T	GS4 ^e^	5.96, 6.37	O2	2f	6.75	2f ${}^{g}$	5.75	2e	5.50
4K9U	GS5 ^e^	5.84, 7.89	O2	S	6.75	2f	6.25	2e, 2a	5.75 ${}^{d}$

**Table 2:** Location of the four major access channels of CYP3A4. ${}^{a}$ According to the authors of [60,69]: C (closed conformation), O1 (conformation opened, in block 1), O2 (conformation opened, in block 2; opening of O2 is larger than opening of O1). ${}^{b}$ The triangulations of the channels performed with CCCPP can be described with lengthy technical details that we consider to be non essential in this paper, such as the lining atoms and residues: see these latter in Table 3.10 in [69]. ${}^{c}$ Substrate recognition sites (SRS), according to the authors of [70]. ${}^{d}$ Depending on the ligand size, channel 2f can offer a larger opening than channel 2a.

Channel	Block	Conformation ${}^{a}$	Exit Location	Exit Location	SRS ${}^{c}$
			In the CYP ${}^{b}$	Outside the CYP	
2a	1	C, O1, O2	Between F-G and B-C loops	Plasma membrane	1, 2, 3
			and $\beta_{1}$ sheet	(PM)	
2f ${}^{d}$	2	O2	Between helix F/F-G loop	Interface	2, 6
			and C-terminal loop	PM/cytosol	
S	2	O2	Between helices E,F,I	Cytosol	4, 6
			and C-terminal loop		
2e		C, O1, O2	Through B-C loop	Cytosol	1

## Abbreviations

The following abbreviations are used in this manuscript:

CCCPP	Computing Cavities, Channels, Pores and Pockets (software)
CV	Critical Value
CYP	Cytochrome P450
MCP	Minimal Cost Path
MD	Molecular Dynamics
PM	Plasma Membrane
RAMD	Random Acceleration Molecular Dynamics
SMD	Steering Molecular Dynamics
SRS	Substrate Recognition Site
